# Vesicular Galectin-3 levels decrease with donor age and contribute to the reduced osteo-inductive potential of human plasma derived extracellular vesicles

**DOI:** 10.18632/aging.100865

**Published:** 2016-01-09

**Authors:** Sylvia Weilner, Verena Keider, Melanie Winter, Eva Harreither, Benjamin Salzer, Florian Weiss, Elisabeth Schraml, Paul Messner, Peter Pietschmann, Florian Hildner, Christian Gabriel, Heinz Redl, Regina Grillari-Voglauer, Johannes Grillari

**Affiliations:** ^1^ Christian Doppler Laboratory for Biotechnology of Skin Aging, Department of Biotechnology, BOKU - University of Natural Resources and Life Sciences Vienna, 1190 Vienna, Austria; ^2^ Evercyte GmbH, 1190 Vienna, Austria; ^3^ Department of Nanobiotechnology, BOKU - University of Natural Resources and Life Sciences Vienna, 1190 Vienna, Austria; ^4^ Department of Pathophysiology and Allergy Research, Center of Pathophysiology, Infectiology and Immunology, Medical University of Vienna, 1090 Vienna, Austria; ^5^ Red Cross Blood Transfusion Service of Upper Austria, Austria; ^6^ Ludwig Boltzmann Institute for Experimental and Clinical Traumatology, AUVA Research Center, 1200 Vienna, Austria; ^7^ Austrian Cluster for Tissue Regeneration, Austria

**Keywords:** Galectin-3, extracellular vesicles, bone, aging, osteogenic differentiation

## Abstract

Aging results in a decline of physiological functions and in reduced repair capacities, in part due to impaired regenerative power of stem cells, influenced by the systemic environment. In particular osteogenic differentiation capacity (ODC) of mesenchymal stem cells (MSCs) has been shown to decrease with age, thereby contributing to reduced bone formation and an increased fracture risk.

Searching for systemic factors that might contribute to this age related decline of regenerative capacity led us to investigate plasma-derived extracellular vesicles (EVs). EVs of the elderly were found to inhibit osteogenesis compared to those of young individuals. By analyzing the differences in the vesicular content Galectin-3 was shown to be reduced in elderly-derived vesicles. While overexpression of Galectin-3 resulted in an enhanced ODC of MSCs, siRNA against Galectin-3 reduced osteogenesis. Modulation of intravesicular Galectin-3 levels correlated with an altered osteo-inductive potential indicating that vesicular Galectin-3 contributes to the biological response of MSCs to EVs. By site-directed mutagenesis we identified a phosphorylation-site on Galectin-3 mediating this effect. Finally, we showed that cell penetrating peptides comprising this phosphorylation-site are sufficient to increase ODC in MSCs. Therefore, we suggest that decrease of Galectin-3 in the plasma of elderly contributes to the age-related loss of ODC.

## INTRODUCTION

Regeneration and repair counteract the accumulation of stochastic damage, the balance of which determines the individual aging process of organisms. The regeneration of tissues and organs by stem and progenitor cells is of utmost importance for a long healthspan. However, the capability of stem cells to regenerate tissue by differentiating into specialized cells has been shown to decrease with age. One organ that is notably affected by this loss in stem cell functionality is the skeleton whose peak bone mass starts to decline after skeletal maturation, reached between the late second to the third decade of life in humans. The bone is a highly dynamic organ that is constantly remodeled and maintained by the coordinated activity of bone forming osteoblasts and bone excavating osteoclasts [1]. This balance is particularly important at older age, as too high osteoclast activity versus too few osteoblasts is considered to give rise to lower bone strength. The molecular mechanisms, by which the imbalance is caused in the elderly, are still incompletely understood. However, it is clear, that after skeletal maturation a constant number of MSCs and a reduced number of mature osteoblasts are observed with increasing age [2]. This indicates that the functionality or the osteogenic commitment of MSCs might be impaired. Supportingly, the numbers of pre-osteoblasts, pre-osteoclasts and osteoclasts do not change with age per unit bone length, at least in elderly rats. However, a strong decline of mature osteoblasts has been described [3, 4], as well as impaired osteoblastogenesis in age associated osteoporosis [5]. This supports the hypothesis that impaired osteoblastogenesis contributes to age-related bone loss and loss of mechanical strength.

Several signaling pathways, such as RANK/RANKL and the BMP pathways, are involved in regulating bone remodeling [1]. However, the Wnt-β-Catenin-dependent signaling pathway seems to be of specific importance with regard to osteoblastogenesis. Upon binding of extracellular Wnt-proteins, such as Wnt10b, [6–8] to a Frizzled (Fzd) receptor and the co-receptor lipoprotein receptor-related protein 5 (LRP5) or LRP6 [9], β-Catenin is no longer degraded and translocates into the nucleus where it associates with the transcription factors nuclear T cell factor (TCF) and Lymphoid enhancer factor (LEF), resulting in the expression of osteogenesis-relevant genes, such as the master regulator of osteogenesis, Runx-2 [10].

Since it has been proposed recently that the systemic environment of young versus elderly individuals can influence stem and progenitor cell functionality in different tissues and models, such as bone repair [11], specific focus is put on secreted circulating factors, in particular with regard to extracellular vesicles (EVs), small vesicles released by many if not all cell types [12]. The cargo of EVs, consisting of proteins, mRNAs and non-coding RNAs, including miRNAs [13] [14], is selectively packaged and delivered to specific recipient cells over short and long distances [15-17]. Thus it contributes to cell-to-cell communication [18] in both physiological and pathological processes [19-24], as well as in aging and age-associated diseases [14, 22]. Depending on their biogenesis and the cellular state, three different types of EVs are distinguished nowadays [18]: (I) EVs called “shedding microvesicles”, also referred to as “ectosomes”, which directly shed from the cellular plasma membrane [18]; (II) apoptotic bodies blebbing from the plasma membrane of cells in the late stage of apoptosis; and (III) exosomes which originate from the late endosomal compartments [23, 24]. At present, there are no methods established which allow the full separation of the different types of EVs from each other. For this reason the collective term extracellular vesicles is used hereafter.

In the present study we set out to determine whether circulating factors, in particular human plasma-derived EVs from the elderly, contribute to the age-dependent loss of stem cell functionality. We observed that vesicles isolated from young donors enhance osteoblastogenesis *in vitro* compared to elderly-derived EVs. While searching for factors mediating this donor-age-dependent vesicular effect, we identified Galectin-3 to be enriched in EVs from young individuals. Galectin-3 is a lectin consisting of a conserved carbohydrate-recognition domain (CRD), a collagen α-like domain and a short amino-terminal domain [25]. The collagen α-like and the N terminal domain exhibit 6 predicted phosphorylation sites which were shown to influence Galectin-3's subcellular localization as well as its interaction with other proteins [26]. Indeed, we found that increased levels of Galectin-3 have a positive impact on the osteogenic differentiation capacity of MSCs and that extracellular vesicles enriched in Galectin-3 enhance osteoblastogenesis of MSCs. We elucidated its molecular mechanism of action by showing that this protein protects β-Catenin from degradation and that its Serine-96 (S96) phosphorylation site is crucial to mediate this effect. Finally, we demonstrated that cell-penetrating peptides fused to a 13 amino acid sequence, mimicking Galectin-3's Serine-96 phosphorylation site, are able to enhance osteoblastogenesis.

## RESULTS

### Galectin-3 is low in plasma derived EVs of healthy elderly, correlating with lower osteogenic inductivity

**Figure 1:**
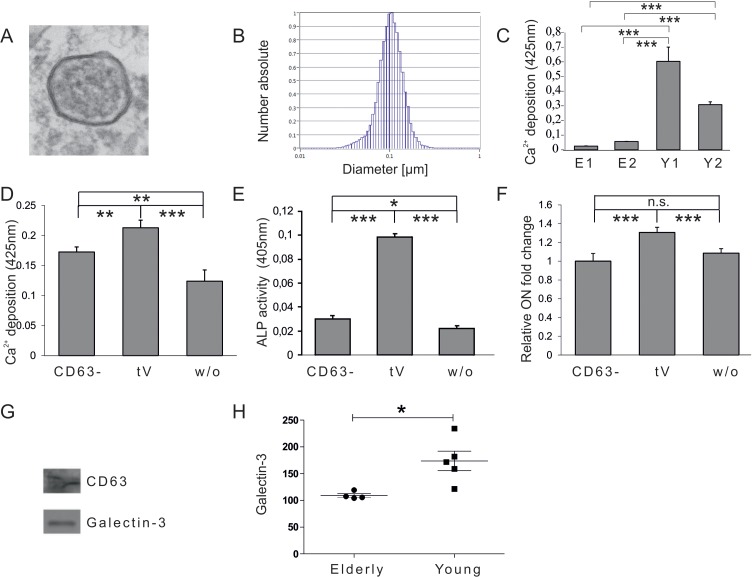
Vesicular impact on osteogenic differentiation capacity of ASCs (**A**) Electron microscopy picture of extracellular vesicles (EVs) isolated by differential centrifugation from human plasma. (**B**) Size distribution of plasma derived EVs analysed by nano tracking. (**C**) Mineralization of ASCs exposed to extracellular vesicles derived from donors older than 55 years (E1, E2) or from donors younger than 25 years (Y1, Y2) was evaluated by Alizarin Red staining. The released dye was quantified by microplate reader at 425nm. Mineralization was significantly increased in ASCs exposed to extracellular vesicles of young donors compared to ASCs exposed to vesicles of elderly donors. (**D**-**F**) Effects of CD63 positive plasma derived extracellular vesicles of young donors on osteogenesis of ASCs. (**D**) Mineralization of ASCs exposed to the CD63^−^ fraction, the total EV fraction (tEV) or of unexposed ASCs was evaluated by Alizarin Red staining. The released dye was quantified by microplate reader at 425nm. Osteogenic differentiation was decreased when cells were exposed to the EV fraction depleted of CD63 positive vesicles. (**E**) Alkaline Phosphatase (ALP) activity was quantified by microplate reader at 405nm. Activity was significantly decreased when cells were exposed to the EV fraction depleted of CD63 positive vesicles. (**F**) Relative fold change of Osteonectin (ON) mRNA levels of ASCs was evaluated by qPCR and normalized to GAPDH. ON mRNA levels were significantly decreased when cells were exposed to the EV fraction depleted of CD63 positive vesicles. (**C**-**F**) ns: not significant, *: p<0.05, **: p<0.01, ***: p<0.001 in comparison to indicated group. Data are presented as mean values ± SD and were statistically analysed using 1-way ANOVA followed by a Bonferroni multiple comparison test, n=4. (**G**-**H**) Plasma derived vesicular Galectin-3 protein levels. (**G**) Detection of Galectin-3 and CD63 protein by Western blot in anti-CD63 immunopurified plasma derived extracellular vesicles. (**H**) Galectin-3 protein levels in extracellular vesicles of donors younger than 25 (Young) or older than 55 years (Elderly) were analysed by ELISA. Vesicular Galectin-3 protein levels significantly decrease with age. Grubbs' analysis identified an outlier in the elderly population (highlighted in red) who was excluded from subsequent statistical analysis.

In order to test whether there is a donor age dependent influence of human plasma derived EVs on the osteogenic differentiation capacity of adipose tissue-derived mesenchymal stem cells (ASCs), ASCs were co-incubated with EVs isolated from the plasma of healthy donors, either younger than 25 years, or individuals older than 55 years, for three days before differentiation was induced. Successful isolation of EVs from human plasma was confirmed by electron microscopy (Fig. 1A) and nano-tracking analysis revealed that isolated extracellular vesicles are approximately 100nm in diameter (Fig. 1B).

ASCs exposed to EVs of young donors exhibited significantly increased osteogenic differentiation capacities as quantified by Alizarin Red staining (Fig. 1C) compared to cells co-incubated with vesicles isolated from elderly donors. Since the EV containing fraction obtained by differential centrifugation might also include co-pelleted protein aggregates or other non-vesicular compounds, the fraction was depleted of EVs by anti-CD63-mediated immunoprecipitation. Subsequently ASCs were co-incubated for 72h with the fraction depleted of CD63 positive vesicles (CD63^−^) or the total extracellular vesicle (tV) containing fraction obtained by differential centrifugation. Unexposed ASCs were included as a control. Upon induction of osteogenesis ASCs co-incubated with the CD63^−^ vesicular fraction of young donors failed to facilitate osteogenic differentiation as efficiently as the total EV containing fraction (tV) as quantified by Alizarin Red staining (Fig. 1D), alkaline phosphatase (ALP) activity assay (Fig. 1E), as well as by qPCR for osteonectin (ON) mRNA (Fig. 1F). This indicates that the pro-osteogenic activity of the total EV containing fraction (tV) mainly, but not exclusively, resides within CD63-positive extracellular vesicles.

In order to identify potential extravesicular proteins that mediate the pro-osteogenic activity of young plasma-derived EVs, we referred to the EV protein database (www.exocarta.org) [29]. One of the factors specifically peaked our interest. Galectin-3, which has already been identified as a component of extracellular vesicles deriving from dendritic cells [30], has also been known to impact on β-Catenin degradation in the context of cancer [31]. We confirmed its presence within the plasma fraction containing CD63 positive EVs obtained by differential centrifugation by Western blotting (Fig. 1G).

To test whether vesicular Galectin-3 levels change with age, we analyzed plasma samples from healthy female individuals. To remove the influence of specific confounding variables which are known to have an impact on plasma Galectin-3 levels, donors were selected according to their age (younger than 25 or older than 55 years), body mass index (BMI < 30), health status (diabetes negative and C reactive protein < 10mg/l), as well as the blood type (blood type A, B or AB). Subsequent analysis of extravesicular Galectin-3 plasma levels by ELISA revealed a significant reduction of Galectin-3 protein levels in healthy individuals older than 55 years compared to donors younger than 25 years (Fig. 1H).

### Galectin-3 is sufficient to modulate osteogenic differentiation

**Figure 2:**
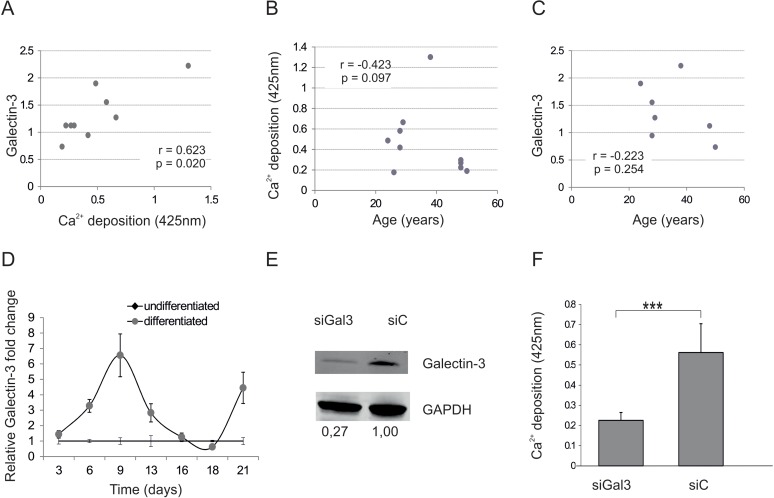

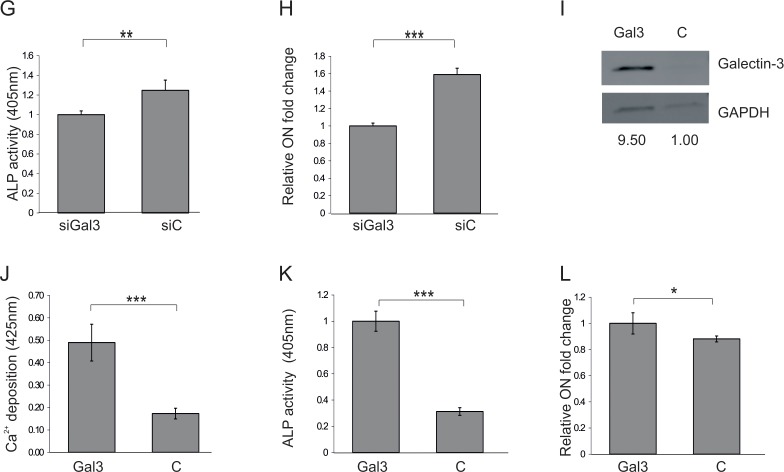
Galectin-3 and osteogenic differentiation capacity (**A**) Spearman correlation of Galectin-3 protein levels before induction of osteogenic differentiation to corresponding mineralization capacity of ASCs. (**B**) Comparison of the mineralization capacity of ASCs from different donors after induction of osteogenesis to the corresponding donors age reveals a not significant trend towards impaired osteogenic differentiation capacity of donors older than 40. N=9 (**C**) Spearman correlation of Galectin-3 protein levels before induction of osteogenic differentiation to the age of the ASC donors. (**D**) Relative fold change of Galectin-3 mRNA levels over a time course of 21 days of differentiated und undifferentiated ASCs was evaluated by qPCR and normalized to GAPDH. Galectin-3 mRNA transcription was significantly increased and peaked at day 9 in ASCs which were induced to undergo osteogenesis (grey squares) compared to undifferentiated ASCs (black dots). N=4 **(E)** Detection of total Galectin-3 and GAPDH protein levels by Western blot in protein lysates derived from ASCs transfected with siRNA against Galectin-3 (siGal3) or with the corresponding non-targeting control (siC). (**F**) Mineralization of siGal3 or siC transfected ASCs was evaluated by Alizarin Red staining. The released dye was quantified by microplate reader at 425nm. Osteogenic differentiation was reduced in siGal3 transfected ASCs compared to control transfected cells. (**G**) ALP activity was quantified by microplate reader at 405nm. Activity was significantly decreased in siGal3 transfected ASCs compared to non-targeting control transfected cells. (**H**) Relative fold change of ON mRNA levels of transfected ASCs was evaluated by qPCR and normalized to GAPDH. ON mRNA levels were significantly decreased in siGal3 transfected ASCs compared to non-targeting control transfected cells. (**I**) Detection of Galectin-3 protein levels normalized to GAPDH protein levels by Western blot in protein lysates derived from ASCs transfected with Galectin-3 overexpression construct (Gal3) or with the corresponding empty vector control (C). **(J**) Mineralization of Gal3 or control transfected ASCs was evaluated by Alizarin Red staining. The released dye was quantified by microplate reader at 425nm. Osteogenic differentiation was enhanced in Gal3 transfected ASCs compared to control transfected cells (C). (**K**) ALP activity was quantified by microplate reader at 405nm. Activity was significantly increased in Gal3 transfected ASCs compared to control transfected cells. (**L**) Relative fold change of ON mRNA levels of ASCs was evaluated by qPCR and normalized to GAPDH. ON mRNA levels were significantly increased in Gal3 transfected ASCs compared to control transfected cells. (**D, F-H, J-L)** *: p<0.05, **: p<0.01, ***: p<0.001 in comparison to indicated group. Data are presented as mean values ± SD and were statistically analysed using unpaired t test, n=4.

To see if Galectin-3 plays a role in osteogenesis, we investigated whether intracellular Galectin-3 protein levels of ASCs might correlate with their differentiation capacity. Therefore, ASCs from 9 different donors aged between 21 and 48 (32.4 ± 9.8 years) were isolated (Table 1 for donor characteristics). In order to examine donor dependent variations in morphology microscopy pictures of ASCs were taken (Fig. S1A). Protein was extracted to quantify Galectin-3 levels by Western blot (Fig. S1B) and cells were tested for their osteogenic differentiation capacity. Interestingly correlation analysis revealed that intracellular Galectin-3 (Fig. 2A) significantly correlates with their Ca^2+^ deposition capability, even better than donor age (Fig. 2B), although several *in vitro* and *in vivo* studies have revealed a reduction in the osteogenic differentiation potential of MSCs isolated from elderly donors, as excellently reviewed by Lepperdinger [32]. Furthermore, intracellular Galectin-3 levels of ASCs do not correlate well with the age of the corresponding donor (Fig 2C), highlighting that Galectin-3 levels might be a better predictor for the osteogenic potential of ASCs *in vitro* than donor age.

**Table 1:** Characterisation of ASC donors

Donor No.	Gender	Age at liposuction [years]	Site of liposuction
**HUF 803**	f	47	femoral
**HUF 846**	f	23	femoral and abdominal
**HUF 851**	f	27	femoral
**HUF 864**	f	37	femoral
**HUF 871**	f	26	femoral
**HUF 887**	f	25	femoral
**HUF 900**	f	48	femoral
**HUF 854**	m	34	abdominal
**HUF 957**	f	44	knee

ASCs isolated from adipose tissue of nine different donors (HUF803, 846, 851, 854, 864, 871, 887, 900, and 957) were used to correlate intracellular Galectin-3 and β-Catenin protein levels to their osteogenic differentiation capacity. The donors gender, year of birth as well as the site of liposuction were cells were isolated from are listed in detail. f=female, m=male

We then analyzed Galectin-3 mRNA levels during the course of osteogenic differentiation (OD). Indeed, Galectin-3 mRNA levels peaked at day 9 after inducing OD compared to undifferentiated cells (Fig. 2D). Subsequently Galectin-3 knock down by siRNA in ASCs was performed in order to see whether Galectin-3 is not only regulated during osteogenesis but also causally influences the differentiation process. Successful knock-down of Galectin-3 was confirmed using Western blot by comparing protein level of siRNA against Galectin-3 and non-targeting control-transfected ASCs (Fig. 2E). ASCs expressing reduced Galectin-3 levels showed a significantly decreased OD capacity as quantified by Alizarin Red staining (Fig. 2F), ALP activity assay (Fig. 2G) and qPCR of osteonectin mRNA (Fig. 2H) compared to non-targeting siRNA control-transfected cells.

Since a lack of Galectin-3 inhibits OD, we tested the effect of Galectin-3 overexpression on OD capacity. By the use of pmaxGFP, a green fluorescent protein (GFP) expression construct, transfection efficiency quantified by flow cytometry was revealed to be higher than 90% (data not shown). Overexpression of Galectin-3 after transfection was confirmed by Western blot (Fig. 2I) and indeed resulted in a significantly increased OD capacity as quantified by Alizarin Red staining (Fig. 2J), ALP activity assay (Fig. 2K) and qPCR on Osteonectin (Fig. 2L).

### Transfer of Galectin-3 from endothelial cells to MSCs by EVs modulates osteogenesis

After elucidating that plasma derived EVs of young individuals are enriched in Galectin-3 protein and are capable to enhance osteoblastogenesis, we set out to test whether such an activity might be mediated by increased Galectin-3 residing in EVs. Firstly, we tested if human umbilical vein cord endothelial cells (HUVEC) derived vesicles can be taken up by ASCs *in vitro*. Early passage HUVECs were transfected by electroporation with pmaxGFP, a GFP overexpression construct. Fig. S2A shows GFP-expressing transfected HUVECs as compared to untransfected cells. Transfection efficiency as analyzed by flow cytometry was 99% (data not shown). 24h post transfection the medium was changed to aspirate remaining plasmids and after a secretion period of 48h EVs were isolated from transfected and untransfected cells. Successful isolation of extracellular vesicles of around 100 nm size from conditioned medium was confirmed by electron microscopy (Fig. S2B) and by nano-tracking analysis (Fig. S2C). High levels of GFP mRNA in EVs deriving from transfected compared to untransfected HUVECs was found (Fig. S2D), while no vesicular GFP protein was detected (Fig. S2E). After co-incubation of ASCs with EVs for 72h, fluorescence microscopy was performed. ASCs exposed to EVs isolated from GFP-expressing HUVECs exhibited an increased intracellular signal of GFP fluorescence (Fig. S2F), as well as intracellular GFP mRNA levels (Fig. S2G), compared to cells exposed to vesicles of untransfected HUVECs, indicating that ASCs are not only targeted by endothelially-derived vesicles, but also translate vesicularly delivered coding mRNAs into proteins.

Furthermore, we investigated whether HUVECs secrete Galectin-3 by EVs and might therefore qualify as a possible *in vivo* source of vesicular Galectin-3 in plasma of young donors. Immunopurification of endothelial cell derived EVs by anti-CD63 antibody coupled beads revealed that Galectin-3 is indeed a component of CD63 positive vesicles (Fig. S2H). Similar to plasma-derived EVs, EVs isolated from *in vitro* aged senescent endothelial cells showed reduced Galectin-3 protein levels compared to EVs of young quiescent cells, as normalized to the number of secreting donor cells (Fig. S2I).

**Figure 3:**
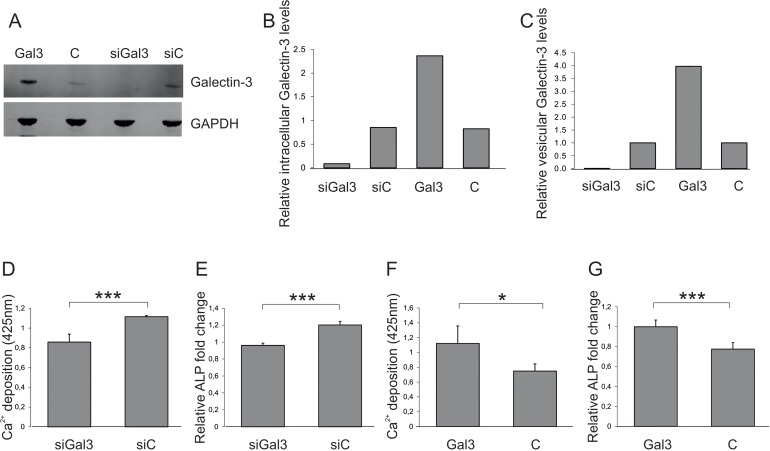
Impact of vesicular Galectin-3 levels on osteogenic commitment of ASCs (**A**) Detection and (**B**) quantification of total Galectin-3 protein levels normalized to GAPDH by Western blot in protein lysates derived from endothelial cells (ECs) transfected with siRNA against Galectin-3 (siGal3), a Galectin-3 overexpression construct (Gal3) or the corresponding controls (siC or C). (**C**) Galectin-3 levels of extracellular vesicles isolated from endothelial cells (ECs) transfected with siRNA against Galectin-3 (siGal3), a Galectin-3 overexpression construct (Gal3) or the corresponding controls (siC or C) were analysed by ELISA and normalized to the number of donor cells (**D-E**) ASCs were exposed to EVs isolated from siRNA against Galectin-3 (siGal3) or corresponding non-targeting control (siC) transfected endothelial cells. Osteogenic differentiation was reduced in ASCs co-incubated with vesicles derived from HUVECs expressing less Galectin-3 (siGal3) as compared to cells exposed to extracellular vesicles of control transfected HUVECs (siC) as evaluated by (**D**) Alizarin Red staining and (**E**) qPCR on ALP mRNA levels normalized to GAPDH. (**F-G**) ASCs exposed to EVs of Galectin-3 expression plasmid (Gal3) or empty vector (C) transfected HUVECS. Osteogenic differentiation was enhanced in ASCs co-incubated with vesicles derived from Galectin-3 overexpressing HUVECs as compared to cells exposed to extracellular vesicles of empty vector transfected HUVECs as quantified by (**F**) was Alizarin Red staining and **(G)** qPCR on ALP mRNA levels normalized to GAPDH. (**D-G**) *: p<0.05, ***: p<0.001 in comparison to control. Data are presented as mean values ± SD and were statistically analysed using unpaired t test, n=4.

Next, the impact of vesicular, endothelially derived Galectin-3 levels on osteogenesis of ASCs was tested. Therefore, HUVECS were transfected with siRNA against Galectin-3, the Galectin-3 overexpression construct or the corresponding controls. Transfection success was confirmed (Fig. 3A) and quantified (Fig. 3B) by Western blot. EVs of transfected cells were isolated after a secretion period of 48h and the reduced or enhanced secretion of vesicular Galectin-3 relative to the corresponding controls was confirmed by ELISA (Fig. 3C). The number of extracellular vesicles was normalized to the secreting cell number and added to ASCs for 72h before OD was induced. ASCs exposed to EVs isolated from siRNA-transfected HUVECS exhibited a reduced OD capacity as quantified by Alizarin Red staining (Fig. 3D) and by qPCR on ALP (Fig. 3E).

On the other hand, ASCs exposed to EVs deriving from Galectin-3-overexpressing endothelial cells exhibited a significantly increased OD capacity as quantified by Alizarin Red staining (Fig. 3F) and by qPCR on ALP (Fig. 3G), compared to cells co-incubated with extracellular vesicles isolated from empty vector control transfected HUVECs. In summary, these data indicate that vesicular Galectin-3 levels have the ability to impact on osteoblastogenesis.

### Serine-96 phosphorylation of Galectin-3 is involved in modulating osteogenic differentiation

**Figure 4:**
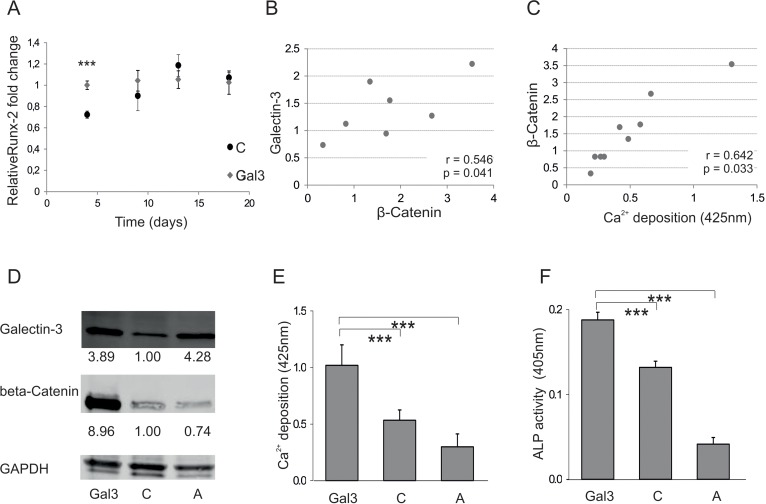
Galectin-3s molecular way of action (**A**) Relative fold change of Runx-2 mRNA levels during osteogenic differentiation over a time course of 18 days. Runx-2 mRNA levels of Galectin-3 overexpressing (Gal3) (indicated in grey squares) or empty vector control transfected cells (C) (displayed by black dots) were evaluated by qPCR and normalized to GAPDH. Runx-2 mRNA transcription was significantly increased at day 4 of differentiation in Galectin-3 overexpressing ASCs (Gal3) compared to empty vector control transfected cells (C) Levels of empty vector control transfected cells are displayed as black dots and data obtained from Galectin-3 overexpressing cells as grey dots. (**B-C**) Spearman correlation of β-Catenin protein levels before induction of osteogenic differentiation to (**B**) intracellular Galectin-3 levels or **(C)** corresponding mineralization capacity of ASCs. (**D**) Overexpression of Galectin-3 wild type (Gal3) and Serine-96 to Alanine (A) mutant compared to empty vector control transfected ASCs (C) was confirmed by Western blot. Galectin-3 as well as β-Catenin protein levels have been normalized to GAPDH (**E-F**) ASCs overexpressing the Galectin-3 mutant (A) showed a significant reduction in their osteogenic differentiation capacity compared to Galectin-3 wild type (Gal3) overexpressing cells as analysed by (**E**) Alizarin Red S staining (**F**) ALP activity assay and (**G**) qPCR on Osteonectin. (**H-J**) ASCs were either untreated (untreated) or exposed to cell penetrating peptides fused to an amino acid sequence which is either mimicking the Serine-96 phosphorylation site of Galectin-3 (Gal3 peptide) or a peptide having all potential phosphorylation sites mutated to Alanine (Mutated). (**H**) Bright field (BF) and fluorescence microscopy in order to detect Bisbenzimide (HÖCHST) stained DNA as well as Fluorescein (FAM) tagged cell penetrating peptides in ASCs exposed to peptides (peptides) or untreated cells (untreated). Fluorescence microscopy reveals an uptake of these Gal3 peptides by ASCs upon co-incubation for 24 hours compared to untreated and Mutated peptide treated cells. (**I-J**) ASCs exposed to peptides for 24h before induction of osteogenesis exhibit a significant increased osteogenic differentiation capacity as evaluated by **(I)** Alizarin Red S staining and (**J**) ALP activity assay as compared to untreated cells. (**A, E-G, I, J**) *: p<0.05, **: p<0.01, ***: p<0.001 in comparison to corresponding control. Data are presented as mean values ± SD and were statistically analysed using (**A, I, J**) unpaired t test or (**E-G**) 1-way ANOVA followed by a Bonferroni multiple comparison test, n=4.

In order to gain insight into the molecular mechanism of Galectin-3-meditated pro-osteogenic activity, we followed the expression of Runx-2. Since this protein is known to be a key transcription factor in OD, the effect of Galectin-3 overexpression on Runx-2 transcription was tested. Galectin-3-overexpressing cells exhibited an earlier induction of Runx-2 when compared to empty vector control-transfected cells, even though at later time points the levels converge to similar amounts (Fig. 4A). In addition, intracellular β-catenin of ASCs (Fig. S3A), necessary to induce Runx-2 expression, was observed to correlate significantly with intracellular Galectin-3 levels (Fig. 4B), as well as with the osteogenic differentiation potential of ASCs (Fig. 4C). Recently, Galectin-3 has been found to be phosphorylated at S96 by GSK3β, a site that might be structurally similar to the phosphorylation site of β-Catenin, which plays an important role in the degradation of β-Catenin. Therefore, we hypothesized that Galectin-3 might compete with β-Catenin for GSK3β mediated phosphorylation. Consequently, high levels of intracellular Galectin-3 might lead to hypophosphorylation of β-catenin, stabilizing it and promoting osteogenic differentiation. Therefore, we compared overexpression of a S96A site-directed mutant of Galectin-3 to wild-type Galectin-3 in ASCs. Indeed, overexpression of the S96A Galectin-3 mutant led to significantly reduced β-Catenin levels (Fig. 4D). In consequence, osteogenic differentiation of cells expressing the S96A Galectin-3 mutant was dampened in comparison to wild-type Galectin-3 expressing ASCs as quantified by Alizarin Red staining (Fig. 4E), ALP activity assay (Fig. 4F) and qPCR for ON (Fig. 4G). In order to test if the functional domain containing the phosphorylation site S96 has a positive effect on osteogenesis, we exposed ASCs to a cell-penetrating peptide TAT fused to 13 amino acids of Galectin-3 covering Serine-96 and a Fluorescein (FAM) tag. ASCs were exposed to these peptides (Gal3 peptide) or to a similar peptide, where all potential phosphorylation sites were mutated to Alanine (mutated) as control. Fluorescence microscopy of ASCs exposed to cell penetrating peptides for 24h revealed that the mutated peptide did not cross the cell membrane. Since only the Gal3 peptides were able to efficiently traverse the cell membrane, (Fig. 4H) we were only able to compare to cells not exposed to any peptides. Exposure of ASCs to Gal3 peptides for 24h resulted in a slightly, but significantly enhanced osteoblastogenesis as quantified by Alizarin Red staining (Fig. 4I) and ALP activity assay (Fig. 4J).

## DISCUSSION

Nowadays stem cell based therapies, which make up an important part of the organism's repair system [33], are thought to have a great therapeutic potential and are already used in clinics [34–36]. Therefore, understanding the biology and the aging process of the systemic environment and its influence on stem and progenitor cells is of particular importance in order to identify factors that impact on regenerative power especially in the case of the elderly.

Reduced commitment of MSCs towards the osteogenic lineage with age has already been reported [4]. In addition, the work of Föger-Samwald and colleagues highlights that impaired osteogenesis also contributes to age-related osteoporosis [5]. Taken together, these investigations suggest that the reduced replacement of specialized bone forming cells indeed contributes to a decline in tissue functionality *in vivo*.

Besides several known intrinsic factors which have been demonstrated to impact on stem cell functionality [37], first indications that extrinsic factors of the systemic or local environment might also contribute to this loss have been provided. Evidence accumulates that cell based therapies are less efficient in the elderly compared to young hosts [38]. Secondly, parabiosis experiments connecting the circulation of elderly and young mice revealed that factors within the circulation impact on the regenerative capacity of the animal [39–42], in particular on bone repair [11], and finally that embryonic stem cells were not able to efficiently persist in the tissue of elderly individuals compared to young ones [43].

Taken together, these studies indicate that environmental factors impact on stem cell functionality, as well. However, only a few secreted factors, such as estrogen [44] or growth hormones [45], are known to contribute to the decline in bone anabolism caused by the aged systemic environment. Influential secreted factors might be provided by extracellular vesicles (EVs) since they are detectable in most body fluids [18], such as the blood [46], and they were shown to protect their cargo from degradation [18]. In addition, EVs were recently demonstrated to contribute to cell-to-cell communication in physiologic, as well as pathologic conditions [14]. Therefore, the effect of EVs isolated from the plasma of young or elderly donors on the differentiation capacity of ASCs was investigated. This study indicates that extracellular vesicles have a donor age-dependent influence on the osteogenic differentiation capacity of ASCs. While searching for vesicular factors that contribute to the pro-osteogenic activity of EVs isolated from the plasma of young individuals, we found that Galectin-3 is enriched in plasma derived vesicles of young donors and that human vesicular Galectin-3 plasma levels decrease with age.

Galectin-3 was already reported to play an important role in cartilage [47] and adipose tissue remodeling [48-49], suggesting that it might be important in stem cell commitment. In regard to bone remodeling, the presence of Galectin-3 was reported to inhibit the formation and maturation of a murine osteoclast precursor cell line *in vitro* [50]. Considering anabolic bone formation, Galectin-3 was confirmed to be important in the late stage of osteoblast maturation [51] and that its expression is mediated by Runx-2 [52]. While we also observed intracellular Galectin-3 upregulation in MSCs during OD at a later time point (day 9), our data also suggest that Galectin-3 does not only play a role in the late stage of osteoblast maturation, but that Galectin-3 transferred to mesenchymal stem cells even before induction of OD has a pro-osteogenic effect. This effect is independent of the transfer of Galectin-3 via extracellular vesicles since ectopic expression in ASCs, as well as donor-dependent differences in Galectin-3 levels of ASCs before osteogenic differentiation was induced, were shown to impact on differentiation capacity. In consequence of a moderate overexpression of Galectin-3, an early induction of ALP and Runx-2 was observed, suggesting that Galectin-3 acts not only downstream of Runx-2, but also upstream. In particular, we demonstrated that Galectin-3's Serine-96 phospho-rylation site is crucial to mediate its positive effect on osteoblastogenesis followed by enhancing intracellular β-Catenin levels. Finally, we observed that a S96-covering sequence fused to the cell-penetrating peptide (CPP) TAT was able to increase osteo-blastogenesis compared to untreated cells. Unexpectedly the CPP fused to the mutated cargo was not able to cross the cellular membrane most probably due to stereochemical changes of the cargo [53].

Finally, we confirmed that the amount of vesicularly delivered Galectin-3 indeed impacts on osteogenic differentiation capacity of ASCs by the use of endothelial cells as donor cells. We chose endothelial cells for several reasons. Firstly it has been confirmed that some MSCs are pericytes [54–56], and are therefore in close proximity to endothelial cells as well as to the vasculature. Therefore the endothelial secretome might not only contribute to the systemic, but also to the local environment of mesenchymal stem cells. Secondly, we identified endothelial cells as a possible source, as they secrete vesicular Galectin-3 *in vitro*. Thirdly, *in vitro* experiments confirmed that EVs secreted by GFP expressing endothelial cells deliver their species-foreign cargo to human ASCs, showing that these two cell types are indeed able to exchange genetic information by extracellular vesicles. In summary endothelial cells may well qualify as a powerful source of released factors that contribute to the systemic and local environment of MSCs.

In regard to circulating Galectin-3 levels several studies have already reported that Galectin-3 protein levels in body fluids significantly correlate with the donors state of health in several diseases, such as various types of cancer [57–63] including osteosarcoma [64], systemic sclerosis [65], inflammatory diseases [66], Alzheimer's disease [67], Diabetes mellitus, type 2 [68] or chronic heart failure [69]. In addition to the existing studies we would add that circulating vesicular Galectin-3 protein levels are decreased in healthy elderly donors compared to young individuals. This suggests that vesicular Galectin-3 might be a pro-osteogenic factor in the young systemic environment supporting bone formation, which is reduced in the elderly.

Taken together, our studies in combination with previous reports indicate an important role for Galectin-3 during bone remodeling. Increased Galectin-3 levels *in vivo* might lead to an enhanced formation of osteoblasts resulting in an improved bone formation. Reduced vesicular Galectin-3 protein levels might contribute to the negative effect of the aged systemic environment on stem cell biology. Therefore, our findings suggest that Galectin-3 might be a valuable plasma-based biomarker for a systemic environment that does not facilitate osteogenic differentiation, a biomarker for the pro-osteogenic capacity of MSCs, but finally also a target for replacement therapy in the elderly.

## MATERIALS AND METHODS

### Cloning

#### Generation of Galectin-3 expression construct

*Homo sapiens* Galectin-3, transcription variant 1 as trans-fection ready DNA was purchased from Origene. The plasmid consists of the human Galectin-3 DNA and the bacterial expression vector pCMV6-XL4. For cell culture experiments human Galectin-3 DNA was cut from the vector by using the restriction enzyme NotI (Fermentas). Subsequently, the Galectin-3 insert was cloned into the pcDNA3.1 hygro (+) vector. Therefore, the vector was digested by NotI and dephosphorylated by calf intestine alkaline phosphatase (Fermentas). Finally the Galectin-3 was inserted by the use of T4 DNA ligase (New England BioLabs). Sequencing was performed in order to validate the correct sequence of the Galectin-3 expression construct.

#### Site directed mutagenesis

Serine-96 to Alanine (S96A) Galectin-3 expression constructs were created by site directed mutagenesis using the QuickChange Multi Site-Directed Mutagenesis Kit (Agilent Technologies) according to manufacturer's instructions. Sequencing was performed in order to validate the correct sequence of the Serine-96 to Alanine Galectin-3 expression construct.

### Cell culture

#### Human umbilical vein endothelial cells (HUVECs)

Cells were isolated from human umbilical veins. HUVECs were grown in gelatin pre-coated flasks in EGM (Lonza) mixed with 10% fetal calf serum (Sigma) at 37°C, 5% CO_2_ and 95% air humidity. Cells were passaged twice a week at a split ratio of 1:2 to 1:8. For isolation of EVs, contact-inhibited quiescent (PD10) and senescent (PD61) cells were allowed to secrete into FCS depleted HUVECS growth medium for 48 hours.

#### Human adipose-derived stem cells (ASCs)

Sub-cutaneous adipose tissue was obtained by outpatient tumescence liposuction under local anesthesia with patient consent. ASCs were isolated according to Wolbank et al. [27] and cultured in DMEM-low glucose/HAM's F-12 (GE-Healthcare) supplemented with 4mM L-glutamine (Sigma), 10% fetal calf serum (Sigma) and 1ng/mL recombinant human basic fibroblast growth factor (R&D Systems) at 37°C, 5% CO_2_ and 95% air humidity. Cells were passaged once a week at a split ratio of 1:2 to 1:6 according to the growth characteristics.

#### Isolation of extracellular vesicles (EVs)

EVs were isolated by differential centrifugation. Firstly, human plasma was diluted 1:2 with PBS and cell culture supernatants were collected after a secretion period of 48 hours. Diluted plasma or conditioned media were centrifuged at 500g for 15 minutes and at 14.000g for 15 minutes to remove cells and cellular debris. Next it was filtered through a 0.22μm filter (Millipore). Finally, EVs were pelleted by ultracentrifugation at 100.000g for 60 minutes. Depending on the subsequent experiment the pellet was resuspended in TRI Reagent (Sigma-Aldrich), protein loading dye or ASC growth medium. For co-incubation studies, EVs isolated from 1 ml of human plasma or from 4×10^4^ HUVECs were resuspended in 1ml growth medium and added per 24 cell culture dish well containing 4×10^3^ ASCs.

In co-incubation experiments ASCs were exposed for 72h to EVs before osteogenesis was induced.

#### Immunoprecipitation of extracellular vesicles

To prepare magnetic anti-CD63 monoclonal antibody immunoaffinity capture microbeads (Dynabeads^®^ M-270 Epoxy, Invitrogen) the Dynabeads® Antibody Coupling Kit (Invitrogen) was used according to the manufacturer's protocol. Therefore, 30 mg of magnetic Dynabeads were coupled to 100μl of monoclonal CD63 antibody (ab8219 Abcam) or 100μl of corresponding mouse IgG1 isotype control (MA1-10406, Thermo scientific).

Next, the pellet obtained by ultracentrifugation was resuspended in PBS (PAA) and incubated with CD63 antibody coupled Dynabeads for 3 h at 4°C on a tube rotator. In order to obtain the fraction depleted of CD63-positive extracellular vesicles (CD63-), the tube was placed on a magnet and the supernatant was carefully transferred to a new 1.5 ml tube. Subsequently, the supernatants were co-incubated with ASCs or centrifuged once more at 100 000g for 2h. The obtained pellet was resuspended in RIPA buffer for subsequent protein analysis.

#### Transfections

Cells were electroporated using the Neon^®^ transfection system (Life technologies) according to the manufacturer's instructions. Briefly 100 000 ASCs or 700 000 HUVECs were resuspended in 10 or 100μl buffer R (Life technologies). For ASCs, 1μg of DNA or 1μl 10μM siRNA (Dharmacon) were added while in the case of HUVECs, 5μg of DNA were used. Electroporation was performed using 3 pulses of 1400V and 10ms for ASCs, while HUVECs were electoporated by 1 pulse of 1350V and 30ms.

#### Induction of osteogenic differentiation in ASCs

4×10^3^ ASCs were seeded in each of the wells in a 24-well culture dish. 72 hours after seeding, osteogenic differentiation was induced by switching the medium to OD medium consisting of DMEM-low glucose (GE-Healthcare), 10% fetal calf serum (Sigma), 4mM L-glutamine (sigma), 10nM dexamethasone (Sigma), 150μM ascorbate-2-phosphat (Sigma), 10mM β-glycerolphosphate (Sigma), 10nM 1.25 Dihydroxyvitamine D3 (Sigma) and 1 x Primocin (Invitrogen) for up to 3 weeks.

#### Cell penetrating peptides

Peptides (>95% purity) were purchased from Biomatik (Cambridge, Ontario, Canada). Cell penetrating peptides deriving from HIV-TAT [28] were fused to a functional domain of Galectin-3. In particular, peptides were designed exhibiting a fluorescein (FAM) tag, an aminohexanic acid (Ahx) as spacer, a TAT peptide which is able to cross cellular membranes, a GG spacer and the Galectin-3 mimicking domain of interest (aminoacids 89 – 101). 5-FAM-Ahx-GRKKRRQRRRGGYPSSGQPSATGAY-Amidation.

As control, a peptide was designed containing a FAM tag, a AHX spacer, the TAT peptide, as well as the GG spacer. However, in the control all potential phosphorylation sites of the Galectin-3 sequence were mutated to alanine. 5-FAM-Ahx-GRKKRRQRRRGGAPAAGQPAATGAA-Amidation.

4000 ASCs were seeded 24 h before peptides were added. After 1 day of co-incubation osteogenic differentiation was induced.

### Analytical methods

#### Alizarin Red S Staining

For quantification of calcified structures, cells were fixed for 1.5 h in 70% ethanol at −20°C. Cells were washed with PBS (PAA) and stained for 20 minutes with 40mM Alizarin Red S solution (Sigma). Subsequently, the remaining dye was removed by rinsing the cells with PBS. Finally, the residual dye was extracted by 0.1M HCL/0.5% SDS solution. The dye was quantified at 425nm.

#### Alkaline phosphatase (ALP) activity assay

In order to determine the activity of ALP, cells were washed with PBS and lysed by 0.25% Triton X-100. Subsequently a buffer containing 20mM 4-nitrophenyl phosphate disodium salt hexahydrate, 0.5 M 2-amino-2-methyl-1-propanol and 0.2 mM MgCl_2_ were added to each cell lysate and incubated for 20 minutes at room temperature. The reaction was stopped by adding 50μl of 0.2M NaOH and ALP activity was quantified by determining the absorbance at 405nm (620nm ref).

#### Quantitative real-time PCR

Total cellular RNA was isolated using TRI reagent (Sigma-Aldrich) according to the manufacturer's instructions. 7 days after induction of OD the early osteogenic marker alkaline phosphatase (ALP) (fw: GCGCAAGAGACACTGAAATATGC, rev: TGGTGGAGCTGACCCTTGAG) was analyzed, while 14 days after differentiation start the osteogenic marker osteonectin (ON) (fw: ATCTTCCCTGTACACTGGCAGTTC, rev: CCACTCATCCAGGGCGATGTAC) was used to quantify OD. In addition, Galectin-3 (fw: ATGCAAACAGAATTGCTTTAGATT, rev: AGTTTGCTGATTTCATTGAGTTTT), as well as Runx-2 mRNA (fw: CTTCACAAATCCTCCCCAAG, rev: GAATGCGCCCTAAATCACTG) expression was checked at several points in time during the OD process. The mRNA levels of GAPDH (fw: TGTGAGGAGGGGAGATTCAG, rev: CGACCACTTTGTCAAGCTCA) were used as reference and GFP mRNA (fw: TGAGCAAGGGCGAGGAGC, rev: GCCGGTGGTGCAGATGAACT) was analyzed to test for a transfer of genetic information by EVs. Reverse transcription was performed using DyNAmo cDNA Synthesis Kit (Finnzymes) and qPCR was performed using the HOT FIREPol EvaGreen qPCR mix (Biotium) and RotorGene2000 (Corbett).

#### Enzyme linked immunosorbent assay (ELISA)

In order to quantify the amount of Galectin-3 within plasma derived extracellular vesicles, EVs were isolated as described above and resuspended in a mixture of 25% RIPA buffer, 25% sample diluent (ELISA kit) and 50% dH_2_O to lyse the vesicles. Next Galectin-3 human simplestep ELISA (Abcam) was performed according to manufacturer's instructions. Therefore, standards were diluted in a mixture of 25% RIPA buffer, 25% sample diluent and 50% dH_2_O.

#### Western Blot

Proteins from cells or extracellular vesicles were extracted by lysing the samples in sodium dodecyl sulphate (SDS) loading dye which contains 5 % β-Mercaptoethanol and by subsequent sonication (Bioruptor Plus, Diagenode).

Next samples were subjected to 4–15% SDS-polyacrylamide gel electrophoresis (Biorad). Proteins were transferred by semi-dry electroblotting to PVDF membranes (Biorad). Membranes were exposed to antibodies against Galectin-3 (ab2785, Abcam, 1:1000), total β-Catenin (PA5-19469, Thermo Scientific, 1:200) or GAPDH (MA5-15738, Pierce 1:5000). Anti-Rabbit-IR-Dye 800 (LiCor, 1:10000) or anti-mouse-IR-Dye® 680RD Donkey anti-Mouse (LiCor, 1:10000) were used for subsequent detection by Odyseey (Licor) infrared image system (LiCor).

#### Nanoparticle tracking analysis (NTA)

For NTA the NanoSight system was used to determine the size distribution and the concentration of isolated extracellular vesicles. All reagents used for this method were passed through a 0.22μm filter before use. Isolated EVs were resuspended in PBS and measured in several dilutions. PBS was used as negative control.

#### Statistics

Data were statistically analyzed using Student's *t* test, one-way ANOVA, followed by a Bonferroni multiple comparison test and Spearman or Pearson correlation as indicated. Analyses were performed with GraphPad Prism. The tests were two-sided with type 1 error probability of 0.05. Data are presented as mean values ± SD.

Samples were blinded systematically and repetitions of experiments were performed by different operators and methods having unbiased read-outs were chosen.

## SUPPLEMENTARY FIGURES


