# Erratum: Vesicular Galectin-3 levels decrease with donor age and contribute to the reduced osteo-inductive potential of human plasma derived extracellular vesicles

**DOI:** 10.18632/aging.100917

**Published:** 2016-02-28

**Authors:** Sylvia Weilner, Verena Keider, Melanie Winter, Eva Harreither, Benjamin Salzer, Florian Weiss, Elisabeth Schraml, Paul Messner, Peter Pietschmann, Florian Hildner, Christian Gabriel, Heinz Redl, Regina Grillari-Voglauer, Johannes Grillari

**Aging (Albany NY) 2015; 8(1)**: **16-33**.

PMID: 26752347

In this Article, the error was in Figure 4, particularly a half of the data in Figure 4 were missing:

http://www.impactaging.com/papers/v8/n1/pdf/100865.pdf. The correct image is provided below.

**Figure 4:**
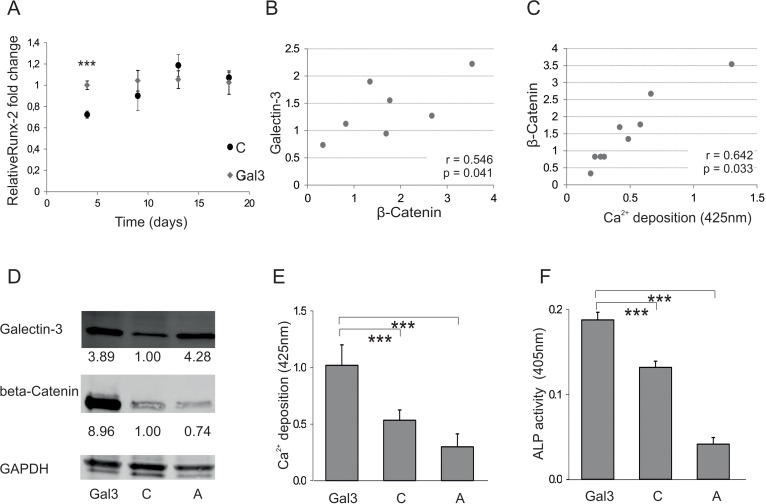

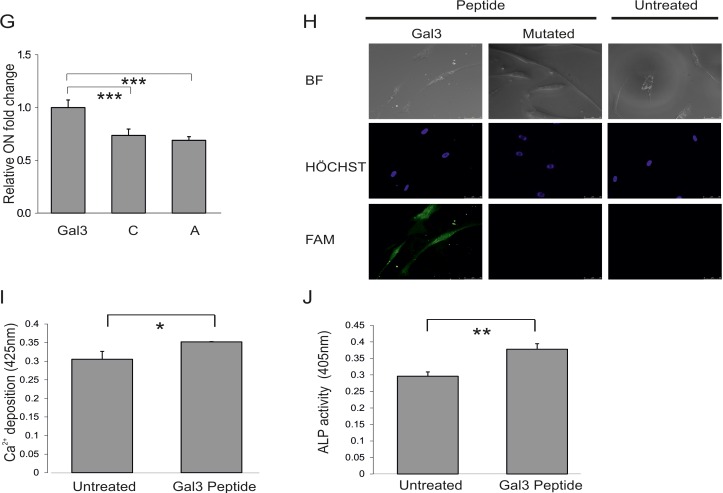
Galectin-3s molecular way of action (**A**) Relative fold change of Runx-2 mRNA levels during osteogenic differentiation over a time course of 18 days. Runx-2 mRNA levels of Galectin-3 overexpressing (Gal3) (indicated in grey squares) or empty vector control transfected cells (C) (displayed by black dots) were evaluated by qPCR and normalized to GAPDH. Runx-2 mRNA transcription was significantly increased at day 4 of differentiation in Galectin-3 overexpressing ASCs (Gal3) compared to empty vector control transfected cells (C) Levels of empty vector control transfected cells are displayed as black dots and data obtained from Galectin-3 overexpressing cells as grey dots. (**B-C**) Spearman correlation of β-Catenin protein levels before induction of osteogenic differentiation to (**B**) intracellular Galectin-3 levels or **(C)** corresponding mineralization capacity of ASCs. (**D**) Overexpression of Galectin-3 wild type (Gal3) and Serine-96 to Alanine (A) mutant compared to empty vector control transfected ASCs (C) was confirmed by Western blot. Galectin-3 as well as β-Catenin protein levels have been normalized to GAPDH (**E-F**) ASCs overexpressing the Galectin-3 mutant (A) showed a significant reduction in their osteogenic differentiation capacity compared to Galectin-3 wild type (Gal3) overexpressing cells as analysed by (**E**) Alizarin Red S staining (**F**) ALP activity assay and (**G**) qPCR on Osteonectin. (**H-J**) ASCs were either untreated (untreated) or exposed to cell penetrating peptides fused to an amino acid sequence which is either mimicking the Serine-96 phosphorylation site of Galectin-3 (Gal3 peptide) or a peptide having all potential phosphorylation sites mutated to Alanine (Mutated). (**H**) Bright field (BF) and fluorescence microscopy in order to detect Bisbenzimide (H&ÖCHST) stained DNA as well as Fluorescein (FAM) tagged cell penetrating peptides in ASCs exposed to peptides (peptides) or untreated cells (untreated). Fluorescence microscopy reveals an uptake of these Gal3 peptides by ASCs upon co-incubation for 24 hours compared to untreated and Mutated peptide treated cells. (**I-J**) ASCs exposed to peptides for 24h before induction of osteogenesis exhibit a significant increased osteogenic differentiation capacity as evaluated by **(I)** Alizarin Red S staining and (**J**) ALP activity assay as compared to untreated cells. (**A, E-G, I, J**) *: p<0.05, **: p<0.01, ***: p<0.001 in comparison to corresponding control. Data are presented as mean values ± SD and were statistically analysed using (**A, I, J**) unpaired t test or (**E-G**) 1-way ANOVA followed by a Bonferroni multiple comparison test, n=4.

