# Supplementary material for: Vesicular Galectin-3 levels decrease with donor age and contribute to the reduced osteo-inductive potential of human plasma derived extracellular vesicles
Source: Aging (Albany NY). 2016 Jan 9;8(1):16–30. doi: 10.18632/aging.100865 (PMC4761711; doi:10.18632/aging.100865)
Supplement: Supplementary file 1 [file aging-08-016-s001.pdf]

## SUPPLEMENTARY FIGURES

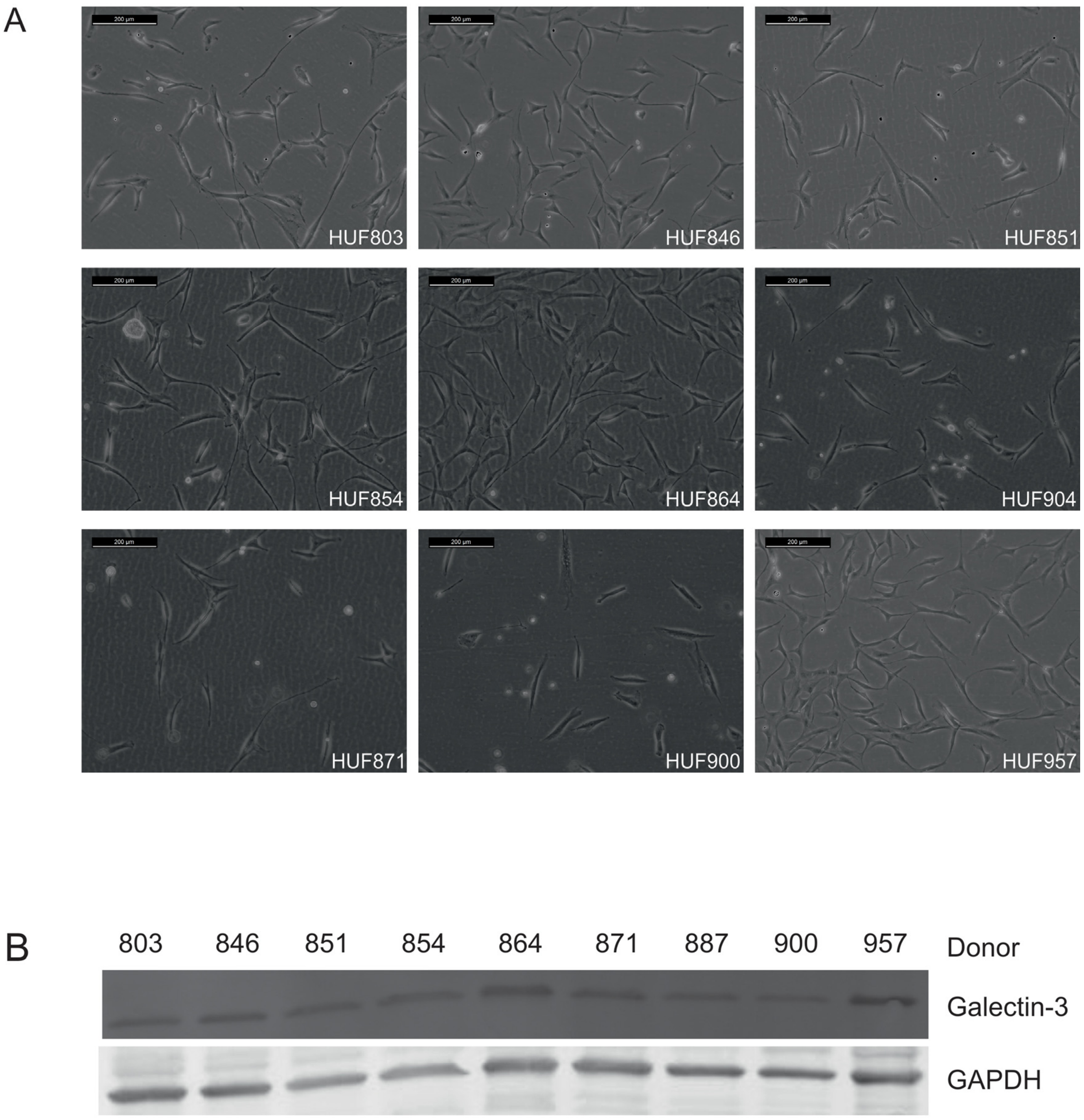


**Figure S1.** (**A**) Bright field microscopy images of ASCs derived from 9 different donors 3 days after toughing. (**B**) Detection of Galectin-3 protein levels normalized to GAPDH by Western blot in protein lysates of ASCs isolated from 9 different donors before induction of osteogenic differentiation.

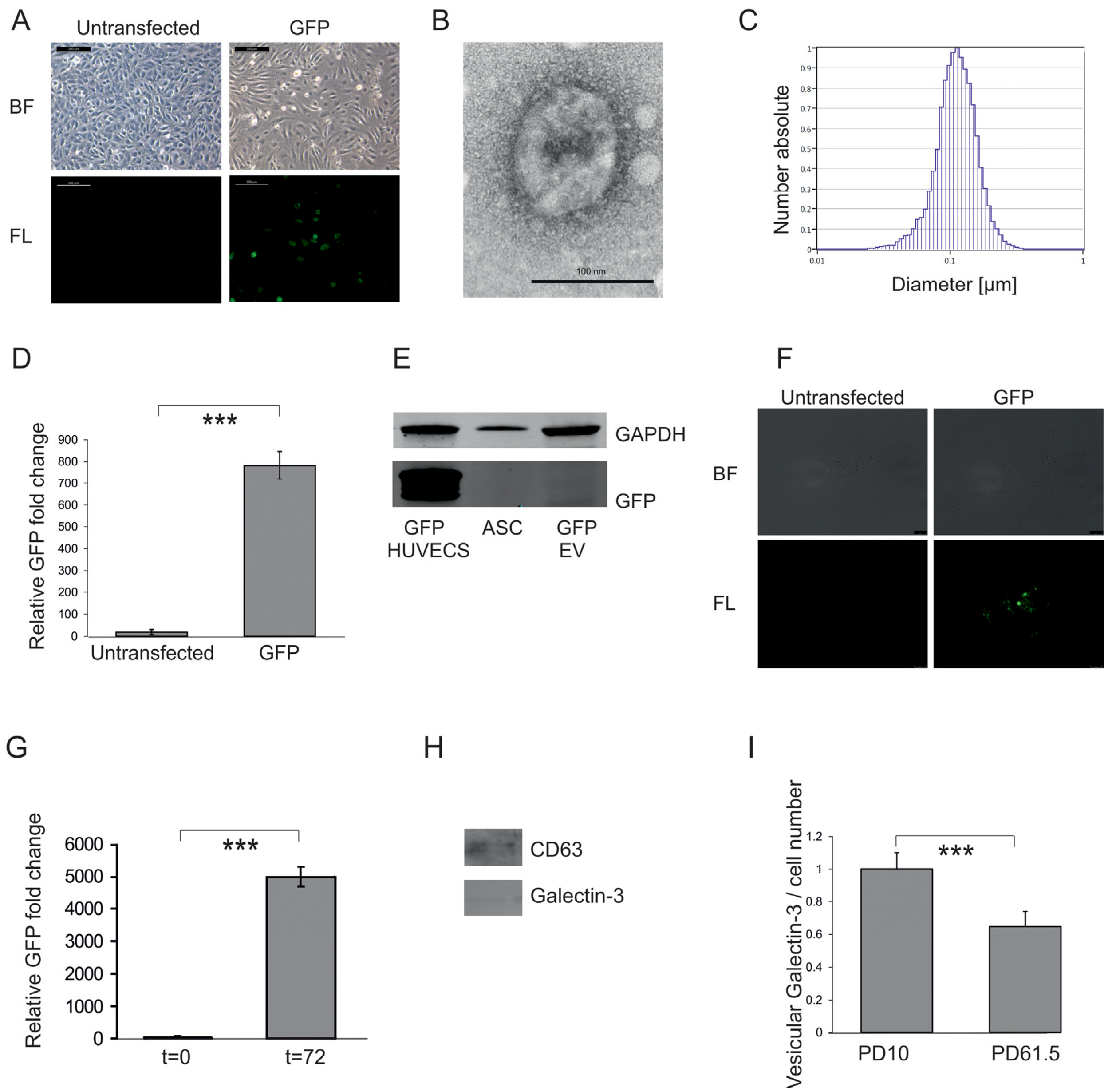


**Figure S2. Endothelial derived vesicles interact with ASCs *in vitro*.** (**A-F**) Transfer of endothelial derived genetic information to ASCs by extracellular vesicles **(A)** Bright field (BF) and fluorescence (FL) microscopy images of endothelial cells transfected with a GFP overexpression construct (GFP) or untransfected cells (untransfected) as control. (**B**) Electron microscopy picture of extracellular vesicles (EVs) isolated by differential centrifugation from conditioned medium of endothelial cells. (**C**) Size distribution of endothelial derived EVs analysed by nano-tracking. (**D**) Relative fold change of GFP mRNA levels within EVs normalized to the number of donor cells was evaluated by qPCR. (**E**) Detection of GFP and GAPDH protein levels by Western blot in protein lysates derived from endothelial cells transfected with GFP overexpression construct (GFP HUVECs) as positive control, ASCs before exposure to EVs as negative control or EVs isolated from GFP HUVECs (GFP EV). (**F**) Bright field (BF) and fluorescence (FL) microscopy images of ASCs after exposure to EVs isolated from GFP expressing HUVECs (GFP) or untransfected cells (untransfected) for 72h. (**G**) Relative fold change of GFP mRNA levels of ASCs was evaluated by qPCR and normalized to GAPDH. GFP mRNA levels were significantly increased in cells after exposure for 3 days to EVs isolated from GFP expressing HUVECs (72h) as compared to the ASCs before co-incubation with EVs (t=0). (**H**) Detection of Galectin-3 and CD63 protein by Western blot in protein lysates of endothelial extracellular vesicles isolated by anti-CD63 immunoprecipitation of conditioned medium. (**I-J**) Galectin-3 levels of extracellular vesicles isolated from early population doubling (PD) quiescent (PD10) or senescent (PD61.5) endothelial cells were analysed by ELISA and normalized to (**I**) the number of secreting donor cells. (**D, G, I**) ***: $p<0.001$ in comparison to control. Data are presented as mean values ± SD and were statistically analysed using unpaired t test, n=4**.**

A

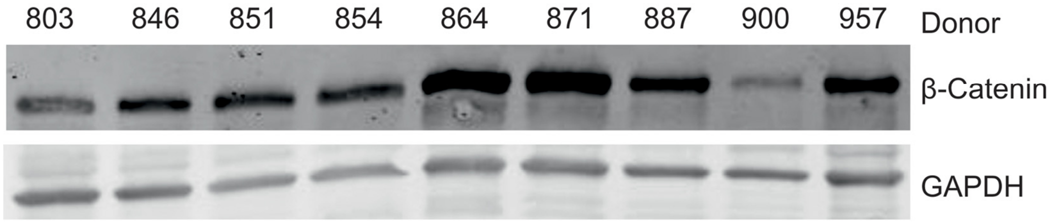


**Figure S3.** (**A**) Detection of β-Catenin protein levels normalized to GAPDH by Western blot in protein lysates of ASCs isolated from 9 different donors before induction of osteogenic differentiation.
